# Neuralgia Treated with Blue Glass

**Published:** 1878-02

**Authors:** 


*Neuralgia Treated with Blue Glass.*—A Baltimorean gives his experience with Blue Glass as follows:

"For several years, until last spring, I suffered from neuralgia in my head and face. I had eminent medical advice, and used internal and external remedies, (quinine and croton oil,) but without permanent relief. About March last I was suffering greatly, when a friend called at my office and told me of a case of relief from blue glass, and advised me to try it. I was ready to try *anything*, and having south windows in my office I ordered twelve half panes of blue glass and had them tacked into the twelve panes of the window next my desk. I have never seen Gen. Pleasanton's book, and, to tell the truth, I had no faith in his theory, but I thought it would be a pretty feature in my office to have the glass in my window, even if it did me no good, and it cost me only about the expense of one or two consultations with my doctor. I thought little of it until I found my pains gone. I am unable to say how many days passed before I was relieved, but I can say that from that time to this I have not had a *single twinge of pain* in my head or face. I have had colds, which always heretofore brought on attacks. I was in the mountains last summer, where I always have had attacks from sudden change of temperature; and even now in this horrid weather I am so free from the affliction that I can hardly realize that I ever suffered from it.

Now, if I am asked if I believe that *blue glass* cured me, I can only answer, as the man in the Bible. 'Whereas I was blind, now I see .' And this reminds me that both my sight and hearing were effected by the attacks of pain, and that both are perfectly restored."

### BIBLIOGRAPHICAL.

